# Drug Delivery Using Nanoparticles for Cancer Stem-Like Cell Targeting

**DOI:** 10.3389/fphar.2016.00084

**Published:** 2016-04-12

**Authors:** Bing Lu, Xiaojia Huang, Jingxin Mo, Wei Zhao

**Affiliations:** ^1^Key Laboratory for Stem Cells and Tissue Engineering, Ministry of Education, Sun Yat-sen UniversityGuangzhou, China; ^2^Department of Histology and Embryology, Zhongshan School of Medicine, Sun Yat-sen UniversityGuangzhou, China

**Keywords:** cancer stem-like cell, induced pluripotent stem cells, epigenetic drugs, nanoparticle, combination therapy

## Abstract

The theory of cancer stem-like cell (or cancer stem cell, CSC) has been established to explain how tumor heterogeneity arises and contributes to tumor progression in diverse cancer types. CSCs are believed to drive tumor growth and elicit resistance to conventional therapeutics. Therefore, CSCs are becoming novel target in both medical researches and clinical studies. Emerging evidences showed that nanoparticles effectively inhibit many types of CSCs by targeting various specific markers (aldehyde dehydrogenases, CD44, CD90, and CD133) and signaling pathways (Notch, Hedgehog, and TGF-β), which are critically involved in CSC function and maintenance. In this review, we briefly summarize the current status of CSC research and review a number of state-of-the-art nanomedicine approaches targeting CSC. In addition, we discuss emerging therapeutic strategies using epigenetic drugs to eliminate CSCs and inhibit cancer cell reprogramming.

## Introduction

While chemotherapy is one of the principal modes of cancer treatment, its effectiveness is limited by drug resistance. The majority of patients with metastatic tumor eventually developing drug resistance and succumbing to their disease. Resistance to chemotherapy can be divided into two categories: intrinsic or acquired. Intrinsic resistance may be due to intratumor heterogeneity that a minor drug resistance subpopulation of cells was present in the original tumor. The cancer stem-like cell (or cancer stem cell, CSC) model provides an explanation for the phenotypic and functional heterogeneity in some type of tumors. It has been proven that developing specific therapies targeted at CSCs can improve the survival and quality of life of cancer patients, especially those with drug resistance. Acquired resistance occurs as a result of genetic and epigenetic alterations that can alter the sensitivity of the drug (Zahreddine and Borden, 2013). The high rate of epigenetic change in tumor cells generates adaptive responses, such as increased therapeutic target gene expression and activation of alternative compensatory signaling pathways. Moreover, epigenetic changes have also been identified as important contributors to the cancer cell dedifferentiation and the non-CSC-to-CSC conversion. Thus drugs targeting the epigenetic regulatory machinery (Biancotto et al., 2010) may be an attractive option to re-sensitize to therapy.

Many strategies have been devised to specifically target CSCs, but with limited success (Krishnamurthy et al., 2015a). But now nanoparticles (NPs) have been designed to specifically and effectively target these hardy cells. A few reviews have discussed the therapeutic CSC targeting strategies that have been employed in using nanocarriers delivering stem cell signaling pathway inhibitors (Abetov et al., 2015; Hong et al., 2015). There is a scarcity of information, and many questions in this area remain unexplored and unaddressed. Here, we summarize the current status of CSC findings, the applications of therapeutic NPs in targeting CSC and potential CSC epigenetic drug targets.

## CSC Theory and Drug Resistance

In CSC theory or hypothesis, CSCs are those cells within a tumor that can self-renew and cause the heterogeneous lineages of cancer cells that comprise tumor (Bandhavkar, 2016). CSCs are highly resistant to conventional chemotherapies owing to various enhanced features, including ATP-binding cassette (ABC) transporter proteins, aldehyde dehydrogenase (ALDH) activity, anti-apoptotic proteins, DNA damage repair and activation of key pro-survival signaling molecules such as Notch and NF-κB (Holohan et al., 2013). However, CSC research faces many challenges. For example, reliable CSC markers have not yet been established, and the stability of the CSC phenotype is still being questioned. Therefore, elucidating CSC origin and tumor cell plasticity will no doubt provide a better understanding on CSC properties and help develop specific therapies targeted at CSCs to improve the survival rate and the quality of life of cancer patients.

### CSC Origins

**FIGURE 1:**
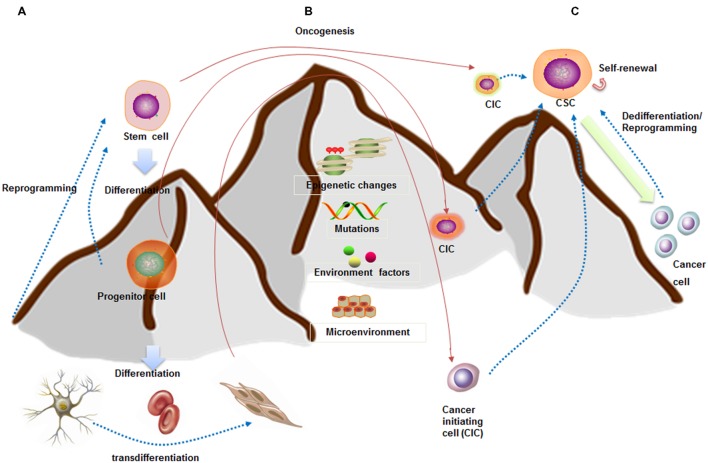
**Stem cell and CSC downhill model (inspired by Waddington’s epigenetic landscape) showing cell fate plasticity. (A)** A stem cell goes through several steps to become differentiated cells (like a ball going downhill). The differentiated cells can be reprogrammed back into a pluripotent state (like hill climbing) or into different lineage cells. **(B)** CSCs are capable of self-renewal and generation of differentiated progeny (like downhill). CSCs may arise from stem cells, progenitor cells, or differentiated cells. **(C)** Increasing evidences indicated the cell fate plasticity between CSCs and non-CSCs. These conversions are dependent on epigenetic regulation, gene expression patterns, as well as interactions with microenvironment.

Whether CSCs arise from stem cells is currently under debate (**Figures 1A,B**). One of the theories argues that CSCs may arise from mutated stem cells because of the similarities between them (Kumar and Kutty, 2012; Ma et al., 2014). These two kinds of cells share similar cell surface markers, naïve phenotypes, signaling pathways (Notch, Wnt, Hedgehog), homing and migrating pathways in several type of cancers, including leukemia and carcinomas of the breast, colon, liver, lung, and pancreas (Vaz et al., 2013; Garcia-Montero et al., 2016). If CSCs arise from the mutated stem cells present in the adult tissue, they could simply utilize the existing stem-cell regulatory pathways to promote their self-renewal (Hope et al., 2004). Taking leukemia as an example, Dutta et al. (2015) inserted the CALM/AF10 fusions gene, preceded by a loxP-flanked transcriptional stop cassette, into the Rosa26 locus. They found that Vav-Cre (VavCre-mediated recombination occurred in most hematopoietic cells) induced pan-hematopoietic expression of the CALM/AF10 fusion gene led to acute leukemia. However, mice expressing CALM/AF10 in the B lymphoid compartment using Mb1-Cre or CD19-Cre did not develop leukemia. These results indicated that the ‘cell of origin of leukemia’ (COL) for CALM/AF10 is a stem or early progenitor cell. There are also similar findings in intestine cancer. Drost et al. (2015) depleted four of the genes (APC, P53, KRAS, and SMAD4) with CRISPR/Cas9 technology in cultured human intestinal stem cells formed organoids. They found that quadruple mutant stem cell organoids can grow with CSC properties *in vitro* and as tumors with features of invasive carcinoma *in vivo*.

Lineage committed progenitor cells can also function like CSCs by means of epigenetics change, mutation, and environmental factors participation, and behave as a cell of origin of disease transformation. Recently, Buechele et al. (2015) generated endogenous MLL-AF9 and MLL-ENL oncogenes through insertional mutagenesis into primary human hematopoietic stem and progenitor cells (HSPCs) derived from human umbilical cord blood. They found the engineered HSPCs induced acute leukemia following transplantation in immunodeficient mice at 16 weeks.

Cancer stem cell may also be the product of dedifferentiation of somatic cells from oncogenic insult, at least in breast cancer, osteosarcoma, and colorectal cancer. For instance, Koren et al. (2015) inserted PIK3CA(H1047R) mutation in the lineage-committed basal Lgr5^+^ and luminal keratin-8^+^ cells of the adult mouse mammary gland evoking cell dedifferentiation into a multipotent stem-like. In osteosarcoma, Di Fiore et al. (2014) found that the ectopic expression of p53-R248W/P72R in MG63 cells promoted cancer stem-like features suggesting that p53 gain of function can be at the root of the dedifferentiation of MG63 cells into CSCs. Moreover, several studies showed that colorectal cancer may arise from more differentiated cells because of constitutive NF-κB activation (Myant et al., 2013). Colorectal cancer also arises from a subpopulation of differentiated quiescent tuft cells positive for DCLK1 upon combined APC deletion and chemical-induced inflammation (Westphalen et al., 2014). Taken together, these studies indicate that CSCs can originate from non-stem cells at least in certain types of cancer. Additionally, two of the fundamental questions are which differentiated cells are susceptible to mutation induced dedifferentiation and how this impacts tumor progression and aggressiveness. We next summarize the dynamic regulation of cancer cell plasticity which is paramount for understanding tumor heterogeneity and CSC origins.

### CSC State Plasticity in Breast Cancer

The traditional hierarchical model believed that CSCs reside in the apices of hierarchies and differentiate into non-CSCs in a unidirectional manner (Chaffer et al., 2011). However, CSCs might be alternatively derived from differentiated cancer cells through different mechanisms, such as tumor niche signals (Pattabiraman and Weinberg, 2014), cellular interactions (Kim et al., 2010), and epithelial to mesenchymal transition (EMT; Schieber and Chandel, 2013) (**Figure 1C**). The breast CSC state plasticity is relatively well studied. It is found that the bulk tumor cells are capable of “dedifferentiating” into CSCs. Guo et al. (2012) showed that differentiated breast cancer cells could be converted into stem-like cells by overexpressing transcription factors SLUG and SOX9. Furthermore, breast CSCs maintain the plasticity to transition between two different phenotypic states: a more proliferative epithelial-like state (ALDH^+^) and a more quiescent but invasive, mesenchymal-like state (CD44^+^/CD24^-^). Transition between the two CSC states is mediated by epigenetic alterations regulated by microenvironment and EMT regulators (Brooks et al., 2015).

In breast cancer, there is now rapidly accumulating evidence showing that the EMT induction and acquisition of CSC characteristics are highly interrelated. The EMT is a highly conserved cellular process that involves normal embryogenesis and tissue repair (Liu and Fan, 2015; Ye et al., 2015). Through an EMT process, tumor cells acquire mesenchymal properties such as increased motility and invasion, which can endow cells with stem-cell like characteristics. Phenotypically, breast CSCs express high CD44 and low CD24 with increased ALDH1 activity. Clinical evidences have shown that the CD44^high^/CD24^low^ cells were more enriched in triple negative (ER/PR- and HER2-) breast cancer (TNBC) and were associated with increased risk for metastases. Many efforts were made to illustrate the molecular links between EMT regulators and breast cancer cell plasticity. In 2013, Weinberg group found that ZEB1, a key regulator of the EMT, can promote non-CSCs (CD44^low^) to enter the CSC state (CD44^high^; Chaffer et al., 2013). Pharmacologic inhibition of the Twist-BRD4 associated EMT suppressed CSC-like properties, and tumorigenicity of basal-like breast cancer cells (Shi et al., 2014). However, early studies focused on xenograft models with ectopic expression of EMT factors that do not exist under physiological conditions. In addition, EMT regulators may have oncogenic functions independently of EMT induction, thus observed phenotypes by gene manipulation may not be exclusively due to EMT. Therefore, more effects are needed to illustrate the importance of EMT in breast non-CSC-to-CSC conversion.

Cancer cell plasticity can also be regulated by tumor niche signals and cellular interactions. A number of studies have demonstrated that a hypoxic niche may play an important role in promoting the breast stem cell pool. Moreover, the relationship between CSCs and their niches can be bi-directional. CSCs can remodel the microenvironmental niches to facilitate survival, stemness, and escape from chemotherapies (Pattabiraman and Weinberg, 2014). Once a thorough understanding of how dedifferentiation contributes to CSC traits has been achieved, we can use this information to facilitate better therapeutic treatments.

### Similarities between Tumor Dedifferentiation and Somatic Cell Reprogramming

**FIGURE 2:**
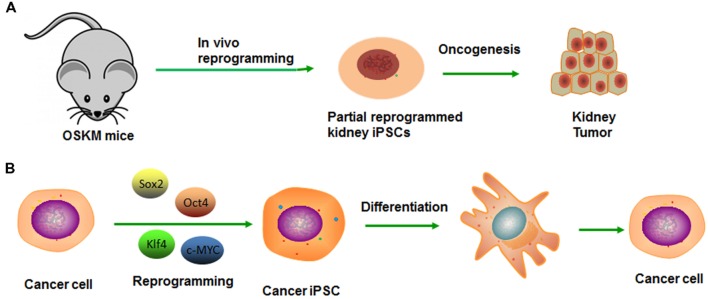
**Modeling cancer with cellular reprogramming technology. (A)** Premature termination of reprogramming leads to tumor development. **(B)** Re-differentiation of cancer cell-derived iPSC can recapitulate the progression of human cancer development.

Induced pluripotent stem cell (iPSC) technology provides a high efficiency and accessible technique to reprogram differentiated cells into a pluripotent state (Takahashi and Yamanaka, 2006). A number of similarities exist between the processes of reprogramming and tumor dedifferentiation (Semi et al., 2013). First of all, although cells may appear phenotypically pluripotent in certain experiments, premature or inaccurate reprogramming at specific gene loci may generate undesirable cells, even be tumorigenic (**Figure 2A**). Second, the reprogramming factors (Oct4, Sox2, Klf4, and c-Myc) also play important roles in tumorigenesis. Exactly as Oct4, the transcription factor which also contributes to premalignant carcinoma *in situ*, as well as high expressing in blastocyst and cancer cell cDNA but low expressing in normal tissue cDNA (Vazquez-Martin et al., 2011). Epithelial dysplasia in mouse cells that induced expression of Oct4 provide further evidence for the important role of stem cell genes in carcinogenesis (Beltran et al., 2011). Similarly, the expression Nanog in Oral squamous cell carcinoma (OSCC) indicates a poor prognosis, as well as nasopharyngeal carcinoma if co-expressed with Oct4 (Li et al., 2013). Third, it was demonstrated that several signaling pathways impacted the reprogramming process by modulating small molecules such as Wnt-β-catenin. Several studies have shown that Wnt/β-catenin pathway activation is associated with poor outcomes in patients with high-grade serous ovarian cancer (HGSOC; Nagaraj et al., 2015). Wnt/β-catenin signaling is activated in ovarian CSCs, and inhibition of β-catenin potently sensitized cells to cisplatin and decreased CSC tumor sphere formation (Nagaraj et al., 2015).

Generation of cancer-specific iPSCs provides a valuable experimental platform to model oncogenesis like CSC and a way to evaluate its potency. After reprogramming, the pluripotency network can reduce aggressive cancer phenotype, which allows early tissue differentiation and development sufficiently (Kim and Zaret, 2015). The cells can re-acquire cancer phenotype along with the re-differentiation, from which the lineage was derived (**Figure 2B**). On this basis, Kumano et al. (2012) reported that the iPSCs derived from a chronic myelogenous leukemia (CML) patient were capable of re-differentiating into hematopoietic cells effectively and recuperating sensitivity to therapeutic drug-imatinib, which reiterated the pathophysiological features of the initial disease (Kumano et al., 2012). Similarly, Jan E. Carette reprogrammed a CML-iPS cell line that can derive three germ layers in the process of teratoma formation (Carette et al., 2010). The loss of the CML phenotype in CML-iPS cells and the expression of pan-hematopoietic marker CD45, stem cell marker CD34 and pan-T cell marker CD43, indicating recover of differentiation potential. However, unlike the parental cell line that was strictly dependent on BCR-ABL oncogene signaling, the CML-iPS cell that had lost this dependency will become resistant to the BCR-ABL inhibitor imatinib. In the same way, Moore et al. (2015) established the Ewing’s sarcoma (EWS)-iPS cells, and the hierarchical cluster analysis demonstrated patterns of global methylation similar to other ES and iPS cell lines. However, the EWS-derived iPS cells may have been only partially reprogrammed if the loss of DNA methylation is not achieved. Despite the clones possessing many of the prototypical pluripotent markers and phenotypic features, they fail to fully recapitulate the developmental potential as they do from non-malignant tissues. Whether it is a consequence of deficiencies in the reprogramming method or an oncogene-mediated blockade of the proper coordinated epigenetic alterations, remains unknown. Moreover, a differentiated cancer cell can be reprogrammed to recapitulate early-stage cancer and progression for discovering markers, pathways, and therapeutics (Kim and Zaret, 2015). And then, these pluripotent cells can redifferentiate into diverse cells, along with the maintaining of inherited specific epigenetic marks or some “epigenetic memory.” They prefer differentiating into the lineage from which the iPS line was derived. In conclusion, cancer is the outcome of multiple steps and comprehensive factors. Thus, the fate-reversing model can replay the tumor process *in vitro*.

## Nanomedical Strategies For CSC Therapy

Cancer stem cells are highly resistant to standard conventional chemo- and radio-therapies, and remain residual following treatments (Lyakhovich and Lleonart, 2016). The mechanism for high drug-resistance was mediated by diverse cellular processes, such as entering into quiescence state (Li and Bhatia, 2011), enhanced DNA damage repair (Sotiropoulou et al., 2014), rapid drug eﬄux (Saha et al., 2012), over-expression of anti-apoptotic proteins (Vinogradov and Wei, 2012), and detoxifying enzymes (Vinogradov and Wei, 2012). Thus eliminating CSCs has become a popular subject of current investigations. One of the recent approaches to target CSC is the application in the form of molecularly directed nanomedicine, which can control drug delivery and release more efficiently. There have been anticipated results in the early stage of clinical trials that a nanocarrier can target and kill the cellular drivers selectively and will alter the clinical management of cancer fundamentally. NPs can sequester chemotherapeutic drugs at a high concentration and release them within the cancer cell following uptake by CSCs, which potentially overcome such resistance mechanisms. The high target selectivity and internalization will be achieved by targeting ligands on the surface of NPs.

Nanoparticles can effectively inhibit multiple types of CSCs by targeting specific markers (ALDH, CD44, CD90, and CD133) and/or specific signaling pathways (Notch, Hedgehog, transforming growth factor-β (TGF-β), and other key developmental signaling pathways) that have been implicated in the maintenance of the CSC pool of many tumors (Hong et al., 2015).

### Aldehyde Dehydrogenases (ALDH) Marker

Recent studies have demonstrated that high levels of ALDH activity are associated with enhanced tumorigenicity and chemoresistance in many cancers (Li D. et al., 2015), such as cervical cancer (Liu and Zheng, 2013), non-small cell lung cancer (Shao et al., 2014), and melanoma (Yue et al., 2015). Li S.Y. et al. (2015) have developed NPs encapsulating low-dose decitabine that can sensitize CSCs with high ALDH activity [ALDH (hi)] to chemotherapy. The NPs were prepared with biodegradable MPEG-b-PLA, which can easily load small molecule drugs such as doxorubicin (DOX) and decitabine (NPDAC). NP_DOX_ were prepared using single emulsion method, and the average diameter was 88.8 ± 2.7 nm. NP_DAC_ were fabricated using the double emulsion method, resulting in a narrow size distribution (79.8 ± 2.3 nm), and the drug loading (DL) and encapsulation efficiency (EE) of DOX were 5.0 and 51.8%, respectively. The DL and EE of NP_DAC_ were 1.0 and 3.7%, respectively. *In vitro* studies revealed that treatment with NPs loaded with low-dose decitabine combined with NPs loaded with NP_DOX_ better reduced the proportion of CSCs with ALDH (hi) in the mammospheres of MDA-MB-231 cells, and better overcame the drug resistance by ALDH (hi) cells.

### CD44 Marker

The receptor CD44 was strongly expressed by the CSCs; it is a signaling platform that integrates cellular microenvironmental cues with growth factor and cytokine signals. Accumulating evidence indicates that CD44, especially CD44v isoforms, are CSC markers and critical players in regulating the properties of CSCs, including self-renewal, tumor initiation, metastasis, and chemoradioresistance (Yan et al., 2015). Aires et al. (2016) successfully applied novel multifunctionalized iron oxide magnetic NPs (MNPs) with antiCD44 antibody and gemcitabine derivatives for the selective treatment of CD44 positive cancer cells. The results confirmed the selective drug delivery potential of the MNPs by the killing of CD44-positive cancer cells using CD44 negative non-tumorigenic cell lines as control in pancreatic and breast cancers cell lines. MNPs have two advantages compared with other nanoplatforms; they can be used to kill cancer cells through hyperthermia and act as contrast agents in MRI (Aires et al., 2016).

### CD90 Marker

CD90 is a glycosyl phosphatidylinositol-anchored membrane glycoprotein of the immunoglobulin superfamily (Haeryfar et al., 2005), it has been identified as a marker for CSCs such as hepatocellular carcinoma (HCC; Luo et al., 2015), and osteosarcoma (Chen et al., 2015), which are responsible for tumorigenic activity. Luo et al. (2015) isolated CD90^+^ cells from hepatoma carcinoma cell (HCC) lines that exhibited increased tumorigenicity, chemoresistance, tumor invasion, and metastasis. Notch pathway was activated in CD90^+^ cells and researchers found that inhibition of Notch pathway in CD90^+^ CSCs decreased tumorigenicity, cell invasion, migration, and expression of stem cell related genes. Activation of the Notch pathway in CD90^-^ cells induced self-renewal, invasion, and migration. Furthermore, Luo et al. (2015) observed that the CSC features were facilitated by stimulating G1-S transition in the cell cycle phase and inhibited apoptosis mediated by the Notch pathway. Yang et al. (2008) loaded photosensitizers trifluoperazine in anti-CD90 antibody-mediated water-soluble CdSe core nanocrystals to target the CD90^+^ leukemia CSCs specifically; it showed leukemia CSCs sensitized to UV irradiation and leaving apoptotic cell death (Bakalova et al., 2004).

### CD133 Marker

The stem cell marker CD133, also known as prominin-1, is a transmembrane glycoprotein. The protein overexpresses in various cancer types, including metastatic colorectal cancer, ovarian cancer, glioblastoma, and gastric carcinoma. Ni et al. (2015) developed salinomycin-loaded PEGylated poly (lactic-co-glycolic acid) NPs (SAL-NP) conjugated with CD133 aptamers (Ap-SAL-NP). SAL-NP had an average size of 133.4 nm, whereas Ap-SAL-NP had a slightly larger size of 159.8 nm, indicating that the modification of CD133 aptamers increases the size of SAL-NP. The polydispersity index (PDI) of the NPs is smaller than 0.2, suggesting that the size distribution of these NPs is narrow. The proportion of CD133^+^ osteosarcoma cells in the excised tumors was significantly reduced by Ap-SAL-NP treatment compared with salinomycin and SAL-NP, which demonstrated that Ap-SAL-NP has the potential to effectively target and eliminate CD133^+^ osteosarcoma CSCs both *in vitro* and *in vivo* (Ni et al., 2015). More recently, loading chemotherapeutic antitumor drugs and siRNA into Mesoporous silica NPs (MSNPs) which are of thermo/pH-coupling sensitivity and site-specificity, were successfully delivered into CD133^+^ cancer cells in laryngeal cancer mouse mode (Qi et al., 2015).

### Notch Signaling Pathway

Notch signaling, a key regulator of stem cells, frequently sustains activation in many cancers. It often relates to aggressive, evading standards, so that highlighting Notch appears an exciting therapeutic target. The pathway, in principle, can be blocked by γ-secretase inhibitors (GSIs), inhibitory peptides and antibodies, in principle; however, clinical use of Notch inhibitors is restricted by severe side effects. Therefore, conjugated with imagable ligands, MSNPs loaded GSIs system was used to control the delivery of GSIs to target the Notch pathway efficiently. A recent study suggested that inhibition of Notch signaling sharply decreased self-renewal, clonogenic, and the tumorigenic potential of glioblastoma CSCs (Chenna et al., 2012). In addition, inhibition of Notch signaling led to a decrease of the CSC-like subpopulation and increased the susceptibility of CSCs to radiation-induced apoptosis in glioblastomas. Aberrant activation of Notch signaling has been observed in CD133^+^ liver CSC subpopulations when compared with CD133^-^ subpopulations (Zhu et al., 2015).

### Hedgehog (Hh) Signaling Pathway

Similarly, the Hedgehog (Hh) signaling pathway is a critical regulator during early development and regeneration. It is also identified as an important regulator in cell differentiation, growth, and migration. Mutations in different components of sonic hedgehog (Shh) pathway can lead to the development of many cancers including melanoma, medulloblastoma, rhabdomyosarcoma, basal cell carcinoma, breast, lung, liver, pancreas, and prostate cancers (Tang et al., 2012; Verma et al., 2015). Almost currently available Hh small-molecule inhibitors approved for trials for cancer therapy are Smo antagonists. However, clinical application was restricted by their limited binding ability to Smo and poor systemic bioavailability. Using anthothecol which acted via the Sonichedgehog signaling pathway, Verma et al. (2015) developed anthothecol-encapsulated PLGA-NPs (Antho-NPs) to regulate the behavior of pancreatic CSCs. The result shows Antho-NPs significantly reduced the ability of cell proliferation and colony formation, and induced apoptosis in pancreatic CSCs. No effect was observed in normal pancreatic ductal epithelial cells (Verma et al., 2015).

### Transforming Growth Factor-β

Transforming growth factor-β signaling is an important prognostic marker in various types of cancer. While the oncogenic effects of TGF-β in several types of tumors are well-documented, its potential role in CSCs has only recently emerged; selective targeting of TGF-β signaling may be considered as an effective therapeutic strategy for the treatment of various types of cancer. Synergistic treatment with a NP drug delivery system, loading TGF-β signaling pathway inhibitor enhanced tumor penetration and CSCs clearance *in vivo*. Zuo et al. (2016) had constructed cationic lipid-assisted polymeric NPs with siRNA encapsulation using a double emulsion method, and the NPsiRNA showed sustained and sufficient cumulative release of siRNA, Combined siRNA and LY364947, which is a TGF-βR-I inhibitor, act as a penetration enhancer in NPs; the work showed remarkable tumor regression and a notable decrease in CSC frequency (Zuo et al., 2016). Huan Meng developed a nanocarrier, which, used a polyethyleneimine (PEI)/polyethylene glycol (PEG)-coated MSNP for molecular complexation to a small molecule TGF-β inhibitor, LY364947. Because of the high loading capacity and pH dependent LY364947 release from the MSNPs, the platform facilitates systemic biodistribution and retention at the tumor site (Meng et al., 2013).

Collectively, these studies suggest that targeting signaling or markers by various nano-carriers might be an effective therapeutic approach in patients with recurrence following curative surgical resection, as well resistance to chemotherapy.

## Nanoparticle Platforms For CSC-Targeted Drug Delivery

Unlike conventional chemotherapy, NPs have their distinctive and multifunctional properties and various physicochemical structures that carry chemotherapeutic drugs at a high concentration, releases them when arriving at the destination and are uptaken by CSCs. The addition of targeting ligands to the surface of NPs may increase both target selectivity and internalization. In order to accommodate multifarious environment *in vivo*, a huge diversity of NP platform has been developed to contain different sizes, configurations, chemical properties, and biofunctional compositions. In the following section, we will summarize the most widely studied organic and inorganic NPs in current researches (**Table 1**).

**Table 1:** List of NPs targeting CSC through specific markers or signaling pathways.

Type of nanoparticle	Target	Anticancer agent	Type of cancer	Reference
Liposomes	Nucleolin, CD44	F3 peptide, γ-secretase inhibitors (GSIs), Tamoxifen, Paclitaxel	Triple negative breast cancer (TNBC)	Parvani et al., 2015
Micelles	ALDH1A1, CD44	Paclitaxel (PTX), anti-CD44 antibodies, Cetuximab (anti-EGFR), phenformin, gemcitabine, thioridazine (THZ), Twist1 siRNA	Breast cancer, lung cancer	Gener et al., 2015; Krishnamurthy et al., 2015b
Polymeric nanoparticles	ALDH, CD133	Hedgehog pathway inhibitor (HPI)	Brain tumor	Lim et al., 2011; Chenna et al., 2012
Gold nanoparticles	TGF-β, acid-labile hydrazone bond	Doxorubicin	Breast cancer	Sun et al., 2014

### Liposomes

Liposomes were defined as spherical polymeric vesicles consisting of an aqueous core surrounded by one or more concentric phospholipid layers. It shows a wide range of potential application as they are able to carry hydrophilic, hydrophobic, and amphiphilic molecules, as well as are easily manipulated during their production process.

Yang et al. (2008) have developed a novel liposome formulation bearing anti-CD44 antibody to target a bellicose hepatocellular CSC with high tumorigenicity and metastatic potential that over expressed CD44. Doxorubicin were loaded as model drug in targeted liposomes and then injected intravenously into tumor-bearing mice, resulting in a sevenfold higher drug concentration in tumors compared with free DOX, which caused smaller tumor volume. Encouragingly, this effect was achieved by little changes in mouse body mass. Injection of free drug generated similar results in tumor burden but with a significantly high loss of body mass (>30%) in exposed animals. Alternatively, the authors could accomplish tumor imaging and gene therapy at the same time to treat the cancer by using the targeted liposome to carry a triple fusion plasmid, involving gene expression cassettes for red fluorescence protein (RFP), renilla luciferase (Rluc), and a truncated herpes simplex virus thymidine kinase (HSV-TTK) gene. Treatment with the combination of HSV-TTK liposome and ganciclovir (a cytotoxic thymidine kinase substrate) caused a robust increase in tumor-localized apoptosis with trifling effect on normal tissues in tumor-bearing mice.

The example of treatment of CSC in breast cancer was reported by Parvani et al. recently, based on the research that surface nucleolin overexpression could be associated with the identification in highly tumorigenic triple negative breast cancer (TNBC) cells (Parvani et al., 2015). They have developed an F3 peptide-targeted liposomal strategy, targeting cell surface nucleolin, and 100% cell deaths were observed under the proposed link between the stem-like phenotype nucleolin expression in TNBC and F3 peptide-targeted synergistic drug combination, rendering 100% cancer cell death. These findings suggest the potential to abolish the plasticity and adaptability associated with CSC. Ahmad A developed dexamethasone-associated liposomal formulation (DX) that can selectively manipulate glucocorticoid receptor (GR) of cancer cells and release its cargo. Dexamethasone (Dex) is a well-established synthetic ligand for the GR. Due to its structural similarities with cholesterol, Dex was directly incorporated alongside cholesterol and cationic lipid to get a DX liposomal formulation. The resultant liposomal formulation of dexamethasone (Dex)-associated liposome (DX) can encapsulate and deliver the anticancer drug ESC8. The results showed this dual-drug loaded liposomal formulation was able to sensitize and kill highly aggressive and drug-resistive breast cancer stem-cell-like cells, ANV-1 (Ahmad et al., 2016).

### Micelles

Micelles are core-shell NPs with a hydrophobic core and a hydrophilic exterior, which are formed from the self-assembly in aqueous media of lipids or other amphiphilic molecules that range in size from 20 to 200 nm in diameter.

Gener et al. (2015) developed a permanent CSC tagging which permits the identification and separation of CSC from heterogeneous populations by using an ALDH1A1/tdTomato reporter vector. Thereafter, they investigated the efficacy of poly [(D,L-lactide-co-glycolide)-co-PEG] (PLGA-co-PEG) polymeric micelles loaded with paclitaxel (PTX); the MS,PI,ZP of PLGA-co-PEG-PTX-CD44, respectively, are 11.67 ± 0.05 nm, 0.037, –5.62 ± 0.41 mV in day 0, 8.52 ± 0.08 nm, 0.043, –5.88 ± 0.01 mV on day 60. Transmission electron micrographs showed monodisperse micelles with a spherical shape with average diameters that correlated with the hydrodynamic measures obtained by DLS. Stability assays confirmed that this concentration remained constant until day 7, functionalized with CD44 antibodies in breast, and Cetuximab (anti-EGFR) in colon cancer cell lines. The results showed that specific active targeting toward surface receptors enhances the performance of nanomedicines and sensitizes CSC to paclitaxel based chemotherapy.

In another study, Krishnamurthy et al. (2015b) loaded phenformin, capable of eliminating CSCs into micelles via self-assembly using a mixture of a diblock copolymer of poly (ethylene glycol; PEG) and functionalized polycarbonate and a diblock copolymer of PEG and acid-functionalized polycarbonate through hydrogen bonding. The phenformin-loaded micelles (Phen M) were more effective in inhibiting the growth of both lung CSCs (side population cells, i.e., SP cells) and non-SP cells. Interestingly, the same group recently further showed that Phen M in combination with gemcitabine-loaded micelles (Gem M) exhibited higher cytotoxicity against lung CSCs and non-CSCs than Gem M and Phen M alone without inducing toxicity to the liver and the kidney. Similarly, micelle was loaded with thioridazine (THZ), which was reported to kill CSCs, in a combination therapy with DOX to eradicate both breast cancer cells and DOX-resistant breast CSCs (Ke et al., 2014).

Furthermore, micelles can be structurally and chemically modified to respond to various environmental stimuli such as a pH-triggering release behavior of the DOX-loaded mixed micelle (DLMM), which releases faster in acidic media (pH 4.0–6.0). For example, Yu et al. (2016) recently reported a novel triple-layered pH-responsive micelleplex loading siRNA and alkylated cisplatin prodrug for treatment of metastatic breast cancer. Such pH-sensitive, stable, and biocompatible nanocarriers are extremely attractive for biosensing and therapeutic applications of CSCs targeting.

Zhang et al. (2012) developed a novel polymeric micelles simultaneous delivery of paclitaxel and salinomycin. Salinomycin can efficiently reduce the CSC population when compared with paclitaxel. A diblock copolymer of poly(ethylene glycol) and poly(ε-caprolactone; PEG-b-PCL) was used to produce micelles and the drugs were loaded into the micelles separately. To target somatostatin receptors overexpressed in cancer cells, paclitaxel-loaded micelles were further wreathed with octreotide. The range in size of micelles is 25–30 nm, which was noticeably smaller than the abovementioned liposomes, thus their ability to gather in tumors via the EPR effect is increasing. *In vitro* the co-delivery of paclitaxel-loaded and salinomycin-loaded micelles killed MCF-7 cells and their effect was comparable to the free drug combination. Yet, *in vivo* findings on MCF-7 mouse xenografts are interesting. The drugs loaded micelles possessed significantly higher anti-tumor effects than the free drugs. The reason for that could be the enhanced buildup of drug-loaded micelles in the tumor site and prolonged drug release.

### Polymeric Nanoparticles

Polymeric NPs have emerged as a valuable nanotechnology platform for controlled, sustained, and targeted delivery of anticancer agents including small molecular drugs, and macromolecules such as genes and proteins may be the best NPs for long-term therapeutic delivery.

Recently Chenna et al. (2012) have developed a polymeric NP loaded with a small molecule inhibitor, HPI-1 (Hh pathway inhibitor), which was shown to overcome the secondary mutational resistance toward Smoothened antagonists. Hh signaling is believed to be abnormally active in most of the human cancers, and Smo secondary mutation annuls the binding of most of the Hh inhibitors. The group tackle this problem by nanoformulating HPI-1 (NanoHHI) that is a strong antagonist of Gli1 and found that NanoHHI strikingly inhibits the growth of mouse medulloblastoma allografts, which shelter a Smo^D477G^-binding site mutation, together with substantial downregulation of Gli1 mRNA. Nanoformulation of HPI-1 increased its water solubility and systemic bioavailability as well (Chenna et al., 2012). The same group of researcher also verified their studies in an orthotopic model by using NanoHHI to check if the inhibition of Hh signaling in HCC is made. NanoHHI significantly diminished systemic metastases in HCC cell lines both *in vitro* and *in vivo*. Additionally, it also reduced the population of CD133^+^-expressing HCC cells, which were regarded as the tumor-initiating cells.

In a research by Lim et al. (2011), they studied the efficacy of a patented polymer-encapsulated curcumin NP formulation (termed NanoCurc^TM^) for the treatment of brain tumor stem cells. The NP formulation greatly increased the bioavailability of curcumin, and increased rates of cell cycle arrest, apoptosis, and dosedependent decreases in growth and clonogenicity were observed after treatment of four distinct brain cancer cell lines with NanoCurc^TM^. Interestingly, this treatment correlated with a >50% reduction in the CD133^+^ stem cell population in two of the cell lines tested, suggesting that this therapy may have its influence in the CSC fraction of some brain tumors (Lim et al., 2011).

### Gold Nanoparticles

Gold NPs (AuNPs) are composed of self-assembled gold atoms that range in size from 1–150 nm in diameter. Proper utilization of gold as nano-sized particles has been investigated in the recent years with the advancement of nanoscience and technology.

Recently, to decorate the surface of AuNPs with poorly self-interacting polymers, Sun et al. (2014) developed a well-elucidated method to rationally design AuNPs. The NP was coated with DOX via a poly (ethylene glycol) spacer and an acid-labile hydrazone bond that can mediate efficiency DOX delivery to breast CSCs. The DOX-Hyd@AuNPs showed efficient DOX transportation to cancer cells as well as responsive intracellular drug release which can reduce their mammosphere formation capacity and their cancer initiation activity, eliciting marked enhancement in tumor growth inhibition in murine models (Sun et al., 2014). Xiong et al. (2014) reported that AuNPs prevented cisplatin-induced acquired chemoresistance and stemness in ovarian cancer cells and sensitized them to cisplatin. They demonstrated that 20 nm AuNPs inhibited proliferation, angiogenesis, and metastases in a preclinical mouse model of ovarian cancer. Mechanistically, AuNPs prevent cisplatin-induced activation of Akt and NF-κB signaling axis in ovarian cancer cells that are critical for EMT, stem cell maintenance, and drug resistance (Xiong et al., 2014).

Tsai et al. (2013) found that AuNPs could selectively capture TGF-β1 through S–Au bonds which was proved using X-ray photoelectron spectroscopy. The binding between cysteine and disulfides residues resulted in the deactivation of the TGF-β signaling pathway. The bond strength of free cysteine (-SH) on the side chain was slightly less than that of disulfide (S–S), which is attributed to the formation of two S–Au bonds. TGF-β has been implicated as a “master switch” in the induction of fibrosis in many tissues, and plays a key role in the EMT with different isoforms mediating various effects. After getting captured by AuNPs, TGF-β1 undergoes significant conformational changes at both secondary and tertiary structural levels after conjugation to the AuNP surface, which results in the deactivation of TGF-β1 protein (Tsai et al., 2013).

Atkinson et al. (2010) recently found local hyperthermia achieved by gold nanoshells plus radiation can eradicate radioresistant breast CSCs. Using both syngeneic mouse and human xenograft models of triple-negative breast cancer, the same group has demonstrated that a subpopulation enriched in CSCs was more resilient to treatment with six gray of ionizing radiation than the bulk of the tumor cells (Atkinson et al., 2010). In contrast, they found a larger decrease in tumor size without an attendant increase in the percentage of CSCs when treating with local hyperthermia for 20 min at 42°C after ionizing radiation using intravenously administered, optically activated gold nanoshells. After 48 h treatment, cells arose from the tumors treated with ionizing radiation plus hyperthermia demonstrated both a significant reduction in tumorigenicity and a more differentiated phenotype than mock- and ionizing radiation-treated tumors. Therefore, they have confirmed that gold nanoshells plus radiation are responsible for eliminate these CSCs *in vivo* and demonstrated that hyperthermia sensitizes this cell population to radiation treatment.

## Epigenetic Drugs, CSC Targeting, and Nanoparticle

Global epigenetic changes, such as DNA methylation and Histone modifications, have been associated with the de/differentiation of normal stem cells and cancer cells. However, the role of epigenetic drugs on CSCs remains under investigation. Epigenetic regulators that carry out different functions can be thought of as being either “writers,” “readers,” or “erasers” (Schnekenburger et al., 2016). Most drugs have been developed to target “writers” and “erasers.” It is well known that Valproic acid (VPA), a histone deacetylase inhibitor, can potently inhibit tumor growth and induce differentiation. Lee et al. (2015) showed that VPA inhibited the self-renewal abilities of head and neck squamous cell carcinoma (HNSCC) CSCs during two serial passages and reduced the expression of stem cell markers, such as Oct4, Sox2, and CD44 (Lee et al., 2015). Sakaki et al. (2015) reported that the H3K27me3 demethylase JMJD3 inhibitor, GSKJ4 induced cell death, loss of self-renewal, and tumor-initiating capacity of ovarian CSCs. NPs should minimize drug release *in vivo* during blood circulation and trigger intracellular delivery through endocytosis, holding promises for increased efficacy of this class of epigenetic inhibitors.

Furthermore, epigenetic “reader” inhibitors, such as bromodomain and extra terminal protein (BET) inhibitors are delivering a novel promising therapeutic opportunity by directly targeting bromodomain proteins (such as BRD4) that bind acetylated chromatin marks. Dawson et al. (2011) showed that GSK1210151A (I-BET151), a novel small molecule inhibitor of the BET family, has profound efficacy against MLL-fusion leukemic cells. Later, Wyspiańska et al. (2014) reported that a specific BET family bromodomain inhibitor, I-BET151, led to growth inhibition in a human erythroleukemic (HEL) cell line isolated from polycythemia vera patients. Another group also reported that treatment with I-BET151 reduced GBM cell proliferation *in vitro* and *in vivo* (Pastori et al., 2014). Unfortunately, BET inhibitor resistance emerges because of leukemia stem cells as reported by Dawson group (Fong et al., 2015). They used primary mouse HSC immortalized with the fusion protein MLL-AF9 to demonstrate resistance to the prototypical BET inhibitor, I-BET. They further showed that resistance to BET inhibitors is in part a consequence of increased Wnt/β-catenin signaling. Although BET inhibitors have limitations, a combination or nanocarrier strategies may enhance the clinical utility of these targeted therapies.

Epigenetic drugs might be a potential therapeutic strategy in combination with conventional chemo-drugs for drug resistant patients by elimination of CSC traits. For example, decitabine (DAC), which is a DNA hypermethylation inhibitor, is an attractive approach to enhancing the chemotherapeutic response and overcoming drug resistance by CSCs. Li S.Y. et al. (2015) showed that treatment with NPs loaded with low-dose DAC (NPDAC) combined with NPs loaded with DOX (NPDOX) better, and reduced the proportion of CSCs with high ALDH activity in the mammospheres of MDA-MB-231 cells. In another recent study, Unland et al. (2015) showed that the pharmacological inhibition of EZH2, an H3K27me3 methylase, synergistically affects the antitumor activity of the epigenetic compounds 5-Aza-CdR and SAHA. Moreover, Matkar et al. (2015) found that an epigenetic pathway involving MLL2 is important for growth of HER2^+^ cells and sensitivity of the cancer cells to a HER2 inhibitor, lapatinib. These findings indicate the potential therapeutic strategy using MLL2 inhibitor in combination of lapatinib delivered by nanocarrier to treat metastatic breast cancer patients (Matkar et al., 2015).

## Conclusion

There is a significant increase in targeting CSCs with the use of NPs-based drug, protein, or nucleic acid delivery. However, a few of the roadblocks of NPs have not yet been overcome. First, CSC targeting is a tricky science, and success in targeting CSCs *in vitro* might not always translate to success *in vivo*. There are obvious limitations in reaching the CSCs *in vivo* owing to inaccessibility of the entire tumor area and microenvironmental factors, which may be circumvented by using environmentally sensitive NPs (such as pH and reduction potential, among others). Also, it is important to better understand the key characteristics of CSCs in order to target them effectively. For example, CSCs always remain a scarce population within a tumor but are able to repopulate when inoculated in a new environment, indicating that there is some level of organization within the apparent anarchy. Knowing the factors that dictate the fate of CSCs can greatly change the way they are targeted. Second, the field of CSCs is relatively new and there are no well-established protocols to distinguish and separate them. Different researchers use different surface markers and biochemical assays for identification. This should be standardized so that therapeutic outcomes of different nanoplatforms can be cross-compared for accelerating the development of effective therapeutic approaches to overcoming cancer relapse and metastasis. Third, different drugs were often loaded separately into NPs as loading several drugs into the same NP is challenging due to different physicochemical properties of the drugs. Thus, two different NPs of drugs that are synergistic may not actually reach a particular cell together, and this may reduce the effectiveness of the therapy. There is a need to develop multifunctional NPs, which can load multiple drugs simultaneously with high capacity. Finally, the toxicity and long-term effects of nanoformulations need to be studied in depth before they can be used in a clinical setting.

It will be imperative to gain a better understanding of the mechanisms involved in the epigenetic regulation of CSC self-renewal and non-CSC reprogramming. Once the mechanism is understood, it can lead to the discovery of new therapeutic targets and the improvement of current clinical therapeutic strategies. Currently, new therapies in the form of NPs-targeting CSC-specific markers or signaling pathways are available or under investigation. Based on nanomedicine studies mentioned in our review, it is apparent that exploiting nanomedicine in treatment of CSCs can lead to a better outcome for cancer patients. These conclusions warrant future NPs studies aimed at providing accurate therapeutic strategies for cancer patients with higher drug delivery efficiency, more CSC specificity and fewer side effects.

## Author Contributions

BL summarized the literature and wrote the manuscript. XH helped with the manuscript writing and prepared figures. JM provided critical comments and wrote part of the manuscript. WZ supervised all the works and wrote the manuscript.

## Conflict of Interest Statement

The authors declare that the research was conducted in the absence of any commercial or financial relationships that could be construed as a potential conflict of interest.
